# Idiopathic sudden sensorineural hearing loss: effectiveness of salvage treatment with intratympanic dexamethasone or hyperbaric oxygen therapy in addition to systemic steroids

**DOI:** 10.3389/fneur.2023.1225206

**Published:** 2023-08-25

**Authors:** Cinzia Mariani, Filippo Carta, Giulia Catani, Sara Lobina, Valeria Marrosu, Simone Corrias, Melania Tatti, Roberto Puxeddu

**Affiliations:** ^1^Unit of Otorhinolaryngology, Department of Surgery, Azienda Ospedaliero-Universitaria di Cagliari, University of Cagliari, Cagliari, Italy; ^2^Unit of Otorhinolaryngology, Department of Surgery, King's College Hospital London, Dubai, United Arab Emirates

**Keywords:** sudden hearing loss, sensorineural hearing loss, hyperbaric oxygen therapy, steroids, intratympanic steroids

## Abstract

**Background:**

The development of standardized treatments for idiopathic sudden sensorineural hearing loss (ISSNHL) is hampered by uncertainty over the etiology of this disorder. Systemic steroids are historically the primary therapy, with variable hearing outcomes. Over the last two decades, intratympanic steroids (ITS) and hyperbaric oxygen therapy (HBOT) have been proposed as salvage treatments in case of failure of systemic steroids. The present study aims to evaluate the effectiveness of these salvage treatments in addition to systemic steroids.

**Methods:**

We performed a retrospective study on 75 consecutive patients with a diagnosis of ISSNHL who were admitted to the Department of Otorhinolaryngology of our hospital between December 2018 and December 2022. All patients received primary treatment with systemic steroids. In case of slight or no hearing recovery within the 5th day from the beginning of the therapy (T1), a salvage treatment with ITS or HBOT was proposed. Patients were divided into three groups according to the therapy received: systemic steroids (group A), systemic steroids + HBOT (group B), and systemic steroids + ITS (group C). Pure-tone average at 500, 1000, 2000, and 3000 Hz and the mean gain were evaluated at T1 and 3 months after the beginning of the salvage treatment (T2). The hearing recovery was assessed according to the Siegel's criteria.

**Results:**

Sixty-two patients (31 men and 31 women, mean age 56 years) with failure of the primary treatment were definitively enrolled in the study: 34 (54.8%) in group A, 16 (25.8%) in group B, and 12 (19.4%) in group C. The ratio of patients responding to therapy was higher in group A (29.4%) than in groups B (18.75%) and C (16.7%). We did not find any statistically significant difference between groups in terms of mean hearing gain at T2 (17.4 ± 15.4 dB in group A vs. 18.6 ± 21.1 dB in group B and 15.7 ± 14.2 dB in group C, *p* = 0.9).

**Conclusion:**

In our experience, ITS or HBOT associated with systemic steroids, as salvage treatment, did not show significant improvement in hearing outcomes. The evolution of ISSNHL, regardless of the treatment, remains unpredictable.

## Introduction

Idiopathic sudden sensorineural hearing loss (ISSNHL) is defined as a sensorineural hearing loss of 30 decibels (dB) or more over at least three consecutive frequencies that occur within 72 h, with no identifiable cause despite adequate investigation (1).

Hearing loss is typically unilateral (2) and can immediately manifest to its maximum extent or evolve progressively. ISSNHL can occur at any age but most often affects adults, and it is equally distributed among men and women (3–5). The estimated annual incidence is 5–20 per population of 100,000 (3, 5, 6). However, the incidence is underestimated because the spontaneous recovery rate ranges from 32 to 65% (7, 8).

Several etiologies have been proposed to explain ISSNHL including viral infection, intracochlear membrane rupture, vascular disorders, and autoimmune reactions; nevertheless, none of these have been definitively proven (9, 10).

The development of standardized treatments for ISSNHL is hampered by uncertainty over the etiology of this condition. Systemic steroids are historically administered as primary therapy, with variable hearing outcomes (11). Over the last 2 decades, intratympanic steroids (ITS) and hyperbaric oxygen therapy (HBOT) have been proposed as salvage treatments in case of failure of systemic steroids. Their mechanism of action in the treatment of ISSNHL is different: ITS acts mainly by reducing inflammation in the inner ear by the diffusion of steroids through the round window, while HBOT increases intracochlear oxygen tension (12).

Currently, there is no unanimous consensus about the efficacy of these treatments. The interpretation of hearing outcomes and the comparison between studies are impaired by the lack of unanimous criteria for evaluating the efficacy of the different treatments (8).

The present study evaluated the therapeutic effectiveness of salvage treatments with ITS or HBOT associated with systemic steroids.

## Materials and methods

We performed a retrospective study on 75 consecutive patients with a diagnosis of ISSNHL admitted at the Department of Otorhinolaryngology of an Italian institution between December 2018 and December 2022 (Ethics Committee protocol number 2022/5138).

All patients underwent a complete clinical history, physical and audiological examination, blood test analysis, and magnetic resonance imaging to rule out secondary causes of sudden deafness. Patients in whom primary etiology could be found were not included in the present study.

Eligibility criteria included an age of at least 14 years, starting of therapy within 7 days of the onset of symptoms, and the availability of a 3-month follow-up. Pure-tone average (PTA), calculated as the arithmetic mean of the hearing thresholds at 500, 1000, 2000, and 3000 Hz (13) in the affected ear, must have been 40 dB or higher, and the affected ear must have been at least 30 dB worse than the contralateral ear in at least 1 of the 4 PTA frequencies.

All patients received primary treatment with systemic steroids: dexamethasone 0.15 mg/kg/day (maximal dose of 10 mg/day) for 3 days, followed by a half tapering every 3 days. Proton pump inhibitors were administered in addition to steroids to prevent gastrointestinal adverse events.

**Figure 1:**
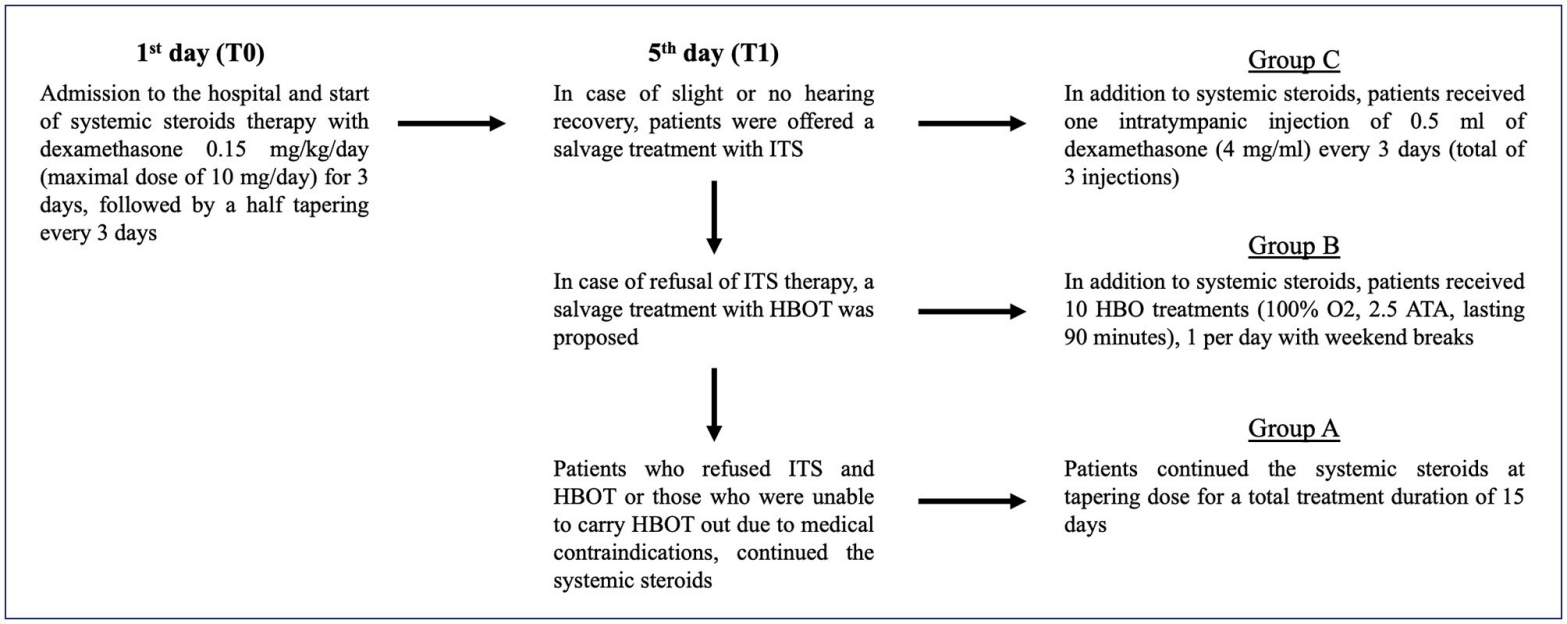
Treatment protocol in patients with ISSNHL.

In case of slight or no hearing recovery within the 5th day from the beginning of the therapy, patients were offered a salvage treatment with ITS as recommended by the American Academy of Otolaryngology, Head and Neck Surgery (AAO-HNS) guidelines (5), and in case of refusal, a salvage treatment with HBOT was proposed. Patients who refused HBOT or those who were unable to carry it out due to medical contraindications continued the systemic steroids alone at a tapering dose. The treatment protocol is summarized in Figure 1.

Pure-tone audiometry was performed pre-treatment (T0) on the 5th day from the beginning of the systemic steroids (T1), and 3 months (T2) after T1, and the mean gain (difference between pre-treatment and post-treatment PTA) was evaluated. The mean gain for each frequency was also evaluated.

Response to therapy was categorized according to Siegel's criteria (14) as follows:

- Complete hearing recovery: PTA better than 25 dB regardless of the size of the gain
- Partial hearing recovery: more than 15 dB of gain and PTA between 25 and 45 dB
- Slight hearing recovery: more than 15 dB of gain and PTA poorer than 45 dB
- No hearing improvement: < 15 dB of gain.

Patients with slight or no hearing recovery at T1 were definitively included in the statistical analysis and were divided into three groups based on the treatment received: systemic steroids (group A), systemic steroids + HBOT as salvage therapy (group B), systemic steroids + ITS as salvage therapy (group C).

In group A, patients continued the systemic steroids at tapering dose for a total treatment duration of 15 days.

In group B, in addition to systemic steroids, patients received 10 HBO treatments (100% O_2_, 2.5 ATA, lasting 90 min), 1 per day with weekend breaks.

In group C, in addition to systemic steroids, patients received one intratympanic injection of 0.5 ml of dexamethasone (4 mg/ml) every 3 days for a total of three injections. The procedure was performed under a microscopic view, with the patient in a supine position with the head turned 45 degrees to the healthy side. Local anesthesia was achieved with 10% lidocaine. After removing the lidocaine solution with suction, an intratympanic injection of a 0.5 ml solution of dexamethasone (4 mg/ml) into the middle ear cavity through the posterior-inferior part of the tympanic membrane was performed using a 25-gauge needle. Following the injection, patients were asked to avoid head movements or swallowing for approximately 10 min.

Patient features including age, sex, presence of vertigo, history of systemic illness such as diabetes mellitus, hyperlipidemia, and cardiovascular diseases, time between the onset of symptoms and therapy, mean PTA at diagnosis, and audiogram shape were evaluated in the three groups.

### Statistical analysis

A power analysis considering a large effect size (0.55) (α = 0.05, power = 0.80) was performed with the statistical software G^*^Power (Version 3.1) and the enrollment of at least 12 patients per group helped to highlight statistically significant differences. Audiological data were presented as mean ± standard deviation. One-way ANOVA, Kruskal–Wallis test, and Fisher's exact test were used for statistical analysis. For all comparisons, a *p*-value of < 0.05 was considered to be statistically significant. All audiological analyses were performed using GraphPad Prism software (GraphPad, San Diego, CA, USA).

## Results

In the period of the study, 75 patients were evaluated for ISSNHL; among them, 13 patients (17.3%) showed a partial or complete hearing recovery within 5 days from the beginning of systemic steroids and were therefore excluded from the statistical analysis.

Sixty-two patients (31 men and 31 women, mean age 56 years, age range 14–81 years) with failure of the primary treatment were definitively enrolled in the study and were divided into three groups based on the treatment received.

The systemic steroids group (Group A) consisted of 34 patients (54.8%), the systemic steroids + HBOT group (Group B) consisted of 16 patients (25.8%), and the systemic steroids + ITS group (Group C) consisted of 12 patients (19.4%). Patient features including age, sex, presence of vertigo, history of systemic illness such as diabetes mellitus, hyperlipidemia, and cardiovascular diseases, time between the onset of symptoms and therapy, mean PTA at diagnosis, and audiogram shape were similar in all three groups, as detailed in Table 1.

**Table 1:** Cohort of patients.

**Variables**	**Group A (Systemic steroids) (*n =* 34)**	**Group B** **(Systemic steroids + HBOT)** **(*n =* 16)**	**Group C (Systemic steroids + ITS) (*n =* 12)**	***p*-value**
Men	15 (44.1%)	12 (75%)	4 (33.3%)	*p =* 0.05
Women	19 (55.9%)	4 (25%)	8 (66.7%)	
Mean age	58	53.9	53	*p =* 0.5
Mean time between onset to treatment (days)	3.5	3.7	3.9	*p =* 0.8
Vertigo	11 (32.4%)	6 (37.5%)	7 (58.3%)	*p =* 0.3
Mean PTA T0 (dB)	83.7 ± 23.5	82.3 ± 25.4	92.7 ± 22.2	*p =* 0.4
**Comorbidities**				
Diabetes	8 (23.5%)	1 (6.25%)	0	*p =* 0.09
Hyperlipidemia	7 (20.6%)	2 (12.5%)	4 (33.3%)	*p =* 0.4
Cardiovascular diseases	14 (41.2%)	3 (18.75%)	2 (16.7%)	*p =* 0.2
**Audiogram shape**				
Ascending	3 (8.8%)	3 (18.75%)	1 (8.3%)	*p =* 0.6
Flat or deaf	15 (44.1%)	7 (43.75%)	6 (50%)	*p =* 0.9
Descending	16 (47.1%)	6 (37.5%)	5 (41.7%)	*p =* 0.8

The mean time between the onset of symptoms and the beginning of systemic steroids in all patients was 3.7 ± 2.2 days. There was no statistically significant difference between groups in terms of the time of starting the therapy (3.5 days in group A vs. 3.7 days in group B and 3.9 days in group C, *p* = 0.8) (Table 1).

The mean PTA of all patients at T1 was 81.8 ± 24.3 dB. Patients of group C showed a worse mean PTA at T1 compared with patients of groups A and B although the difference was not statistically significant (77.4 ± 25 dB in group A vs. 80.1 ± 20.8 dB in group B and 96.5 ± 22.5 dB in group C, *p* = 0.06).

The mean post-treatment PTA of all patients at T2 was 64.4 ± 29.3 dB. Patients of group C showed a worse mean PTA at T2 compared with patients of groups A and B although the difference was not statistically significant (60 ± 31.7 dB in group A vs. 61.5 ± 20 dB in group B and 80.7 ± 29 dB in group C, *p* = 0.1).

We did not find any statistically significant difference between groups in terms of mean PTA gain (17.4 ± 15.4 dB in group A vs. 18.6 ± 21.1 dB in group B and 15.7 ± 14.2 dB in group C, *p* = 0.9) (Table 2).

**Table 2:** Hearing outcomes.

	**Group A (Systemic steroids) (dB ±SD)**	**Group B** **(Systemic steroids + HBOT)** **(dB ±SD)**	**Group C (Systemic steroids + ITS) (dB ±SD)**
PTA T0 (pre-treatment)	83.7 ± 23.5 dB	82.3 ± 25.4 dB	92.7 ± 22.2 dB
PTA T1 (5th day, pre-salvage treatment)	77.4 ± 25 dB	80.1 ± 20.8 dB	96.5 ± 22.5 dB
PTA T2 (3 months after treatment)	60 ± 31.7 dB	61.5 ± 20 dB	80.7 ± 29 dB
Hearing gain (PTA T1-PTA T2)	17.4 ± 15.4 dB	18.6 ± 21.1 dB	15.7 ± 14.2 dB

Hearing gain according to specific frequencies was also analyzed (Table 3). Patients of group B experienced a statistically significant higher mean gain at 4000 Hz than patients of the other groups (*p* < 0.05), while we did not observe statistically significant differences in the recovery of the other frequencies.

**Table 3:** Hearing gain at different frequencies.

	**125** **Hz**	**250** **Hz**	**500** **Hz**	**1000 Hz**	**2000 Hz**	**3000 Hz**	**4000 Hz**	**6000 Hz**	**8000 Hz**
Group A (Systemic steroids) (mean gain ± SD)	13.8 ± 17.9	15.3 ± 18	16 ± 16.8	19.4 ± 18.2	15.1 ± 16.8	14.4 ± 15.5	13.2 ± 16.6	12.5 ± 14.3	13.1 ± 14.3
Group B (Systemic steroids + HBOT) (mean gain ± SD)	15 ± 24.8	12.8 ± 16.7	20.9 ± 20.3	20.6 ± 16.7	20.3 ± 19.8	20.9 ± 18.7	19.7 ± 13.4	15.9 ± 17.1	11.6 ± 15.2
Group C (Systemic steroids + ITS) (mean gain ± SD)	15.4 ± 14.5	20 ± 18	18.75 ± 16.3	19.6 ± 16.7	14.2 ± 13.6	10.4 ± 16.4	8.3 ± 14.1	7.5 ± 12.9	6.7 ± 12.9
*p-*value	0.6	0.5	0.5	0.9	0.6	0.1	< 0.05	0.3	0.3

The ratio of patients responding to therapy (partial or complete hearing recovery according to Siegel) was higher in group A (10 of 34 patients, 29.4%) than that in group B (3 of 16 patients, 18.75%) and group C (2 of 12 patients, 16.7%). The difference between the three groups was not statistically significant (group A vs. group B, *p* = 0.5; group A vs. group C, *p* = 0.5; group B vs. group C, *p* > 0.9).

**Figure 2:**
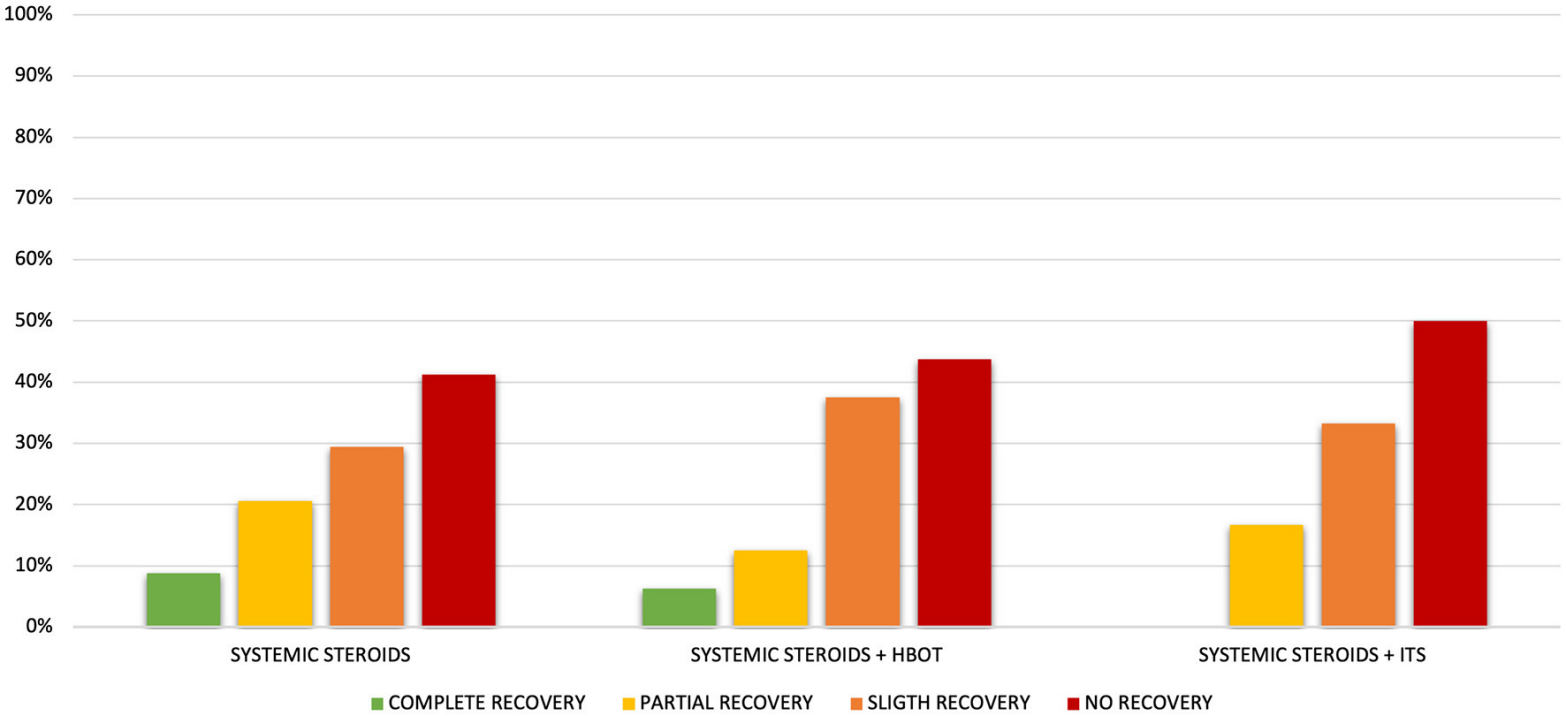
Response to therapy according to Siegel's criteria in each study group.

We have observed a complete hearing recovery in 3 of the 34 patients (8.8%) of group A and in 1 of the 16 patients (6.25%) of group B, whereas no patients of group C experienced a complete recovery although the difference was not statistically significant (*p* = 0.6). Treatment response according to Siegel's criteria is detailed in Table 4 and Figure 2.

**Table 4:** Response to therapy according to Siegel's criteria in each study group.

**Siegel's criteria**	**Group A (Systemic steroids)**	**Group B (Systemic steroids + HBOT)**	**Group C (Systemic steroids + ITS)**
Complete recovery	3/34 (8.8%)	1/16 (6.25%)	0/12 (0%)
Partial recovery	7/34 (20.6%)	2/16 (12.5%)	2/12 (16.7%)
Slight recovery	10/34 (29.4%)	6/16 (37.5%)	4/12 (33.3%)
No recovery	14/34 (41.2%)	7/16 (43.75%)	6/12 (50%)

There were no statistically significant differences between patients who responded to therapy and those who showed slight or no response regarding diabetes, hyperlipidemia, cardiovascular diseases, and audiogram shape. On the contrary, the presence of vertigo proved to be a negative prognostic factor in all three groups, as shown in Table 5.

**Table 5:** Analysis of clinical features related to hearing outcomes.

**Variables**	**Group A**			**Group B**			**Group C**		
	**(Systemic steroids)**			**(Systemic steroids** + **HBOT)**			**(Systemic steroids** + **ITS)**		
	**Response**	**No response**	***p***	**Response**	**No response**	***p***	**Response**	**No response**	***p***
	**(*n =* 10)**	**(*n =* 24)**		**(*n =* 3)**	**(*n =* 13)**		**(*n =* 2)**	**(*n =* 10)**	
Vertigo	0	11 (45.8%)	< 0.05	0	6 (46.1%)	0.25	0	7 (70%)	0.15
**Comorbidities**									
Diabetes	1 (10%)	7 (29.2%)	0.4	0	1 (7.7%)	0.9	0	0	-
Hyperlipidemia	1 (10%)	6 (25%)	0.6	0	2 (15.4%)	0.9	1 (50%)	3 (30%)	0.9
Cardiovascular diseases	2 (20%)	12 (50%)	0.1	1 (33.3%)	2 (15.4%)	0.5	0	2 (20%)	0.9
**Audiogram shape**									
Ascending	1 (10%)	2 (8.3%)	0.9	1 (33.3%)	2 (15.4%)	0.5	1 (50%)	0	0.2
Flat or deaf	5 (50%)	10 (41.7%)	0.7	2 (66.7%)	5 (38.5%)	0.55	0	6 (60%)	0.45
Descending	4 (40%)	12 (50%)	0.7	0	6 (46.1%)	0.25	1 (50%)	4 (40%)	0.9

## Discussion

ISSNHL represents a frightening symptom for the patient and may result in persistent hearing loss with reduced patient quality of life (15).

Until now, more than 60 treatment protocols have been described in patients with ISSNHL, mainly based on etiological hypotheses rather than evidence-based diagnosis. There is still no unanimous consensus on the treatment of choice (16) and on the evaluation criteria for hearing recovery (8). The evaluation criteria reported in the literature range from any improvement to an improvement of 30 dB HL in PTA (8). According to some studies (16, 17), we used Siegel's criteria, which divide the response to therapy into four categories on the basis of hearing gain and PTA, allowing for a more comprehensive evaluation of audiological results.

Over the years, several treatments showed some benefits in restoring hearing; however, as the rate of spontaneous recovery is relatively high and there are no unanimous audiological evaluation criteria, it is difficult to establish which therapy is the most effective (8).

The most widely used treatment for ISSNHL is systemic steroids (10). Steroids were originally implemented in the treatment of ISSNHL because of their anti-inflammatory effect assuming that the cause of sudden deafness was a harmful effect of the immune system on the inner ear in response to viral infection (18, 19). Steroids also have further effects, mainly mediated by activation of the glucocorticoid receptor, such as the reduction of oxidative stress and the reversing of the apoptotic pathway of the injured cochlear hair cells (19). Wilson et al. (18) first stated the efficacy of systemic steroids in the management of ISSNHL, reporting a recovery rate of 61% in their systemic steroid group and 32% in their placebo group. However, the Cochrane review published in 2013 (11) concluded that the evidence supporting the use of systemic steroids is unclear since two of the three included trials demonstrated no significant benefit between steroids and placebo. Contemporary publications on no-treatment or placebo arms in clinical trials are limited since steroid treatment was integrated into the clinical practice guidelines for ISSNHL; therefore, it became highly unethical to randomize a newly diagnosed ISSNHL patient to no treatment or placebo (20). Despite the uncertain balance of benefit vs. harm for steroid therapy based on existing randomized controlled trials (RCTs), there is also unsatisfactory evidence to conclude that the treatment is ineffective (5). Considering the profound impact of ISSNHL on the quality of life, it has been accepted that even a small possibility of a hearing improvement makes systemic steroids a reasonable option (5).

In case of contraindication or failure of systemic steroids, ITS and HBOT have been suggested as first-line or salvage treatments, with variable outcomes reported in the literature.

ITS therapy was proposed as a treatment for ISSNHL by Silverstein et al. (21), and it soon became popular due to the absence of the unfavorable side effects (i.e., diabetes, dysregulation, osteoporosis, or weight gain) of the systemic steroids (17, 22–24). The last guidelines of the AAO-HNS (5) recommend the use of ITS as salvage therapy because most of the studies reported in the literature demonstrated additional hearing improvements with the use of ITS. Indeed, 4 of the 5 RCTs evaluating ITS as salvage therapy found that ITS provided better hearing outcomes than control groups, reporting a hearing improvement in 37–48% of patients (5, 25, 26). Two of these studies (26, 27) administered 40 mg of methylprednisolone in 1 ml of sodium bicarbonate, while the other two studies used 4 and 5 mg/ml of dexamethasone (25, 28). The literature is still inconsistent regarding dose, drug selection, frequency of administration, or the total number of injections (29–31). According to AAO-HNS guidelines (5), dexamethasone or methylprednisolone can be either administered for a maximum of three or four injections. The concentration of intratympanic dexamethasone reported in the literature varies from 4 to 24 mg/ml (5). Some authors recommend the use of higher doses. Alexander et al. (32) compared different concentrations of intratympanic dexamethasone as salvage treatment, reporting a higher improvement rate with a 24 mg/ml dose than with a 10 mg/ml dose (53 vs. 17%, *p* = 0.0382). In our study, patients of the ITS group (group C) underwent three intratympanic injections of 0.5 ml of dexamethasone (4 mg/ml) performed every 3 days. Higher hearing improvements were found at low frequencies, as previously reported by other studies (4, 6). However, none of these patients experienced a complete recovery, and we found a worse PTA at T2 compared to the systemic steroids group (group A) and HBOT group (group B) although the difference was not statistically significant (60 ± 31.7 dB in group A vs. 61.5 ± 20 dB in group B and 80.7 ± 29 dB in group C, *p* = 0.1). It must be considered that the patients of the ITS group had slightly worse pre-treatment PTA, not statistically significant in the present study, which may represent a negative prognostic factor, as previously reported in the literature (16, 33).

HBOT has been used as a treatment for ISSNHL since 1979 (12, 34) with the aim of increasing the partial pressure of oxygen in the blood and then, via diffusion, in the inner ear fluids that nourish the sensory and neural elements of the cochlea (35–38). It is generally recommended that 100% oxygen at 2.0 to 2.5 ATA should be administered for 10 to 20 days, with a 90-min session each day, but there are no HBOT protocols that have been proven to be effective (39–41). Several complications have been described, including barotraumatic lesions (middle ear, nasal sinuses, inner ear, lung, and teeth), oxygen toxicity (central nervous system and lung), confinement anxiety (claustrophobia), and ocular effects (myopia and cataract growth) (38, 41). At the Consensus Conference on Hyperbaric Medicine in 2016 (5, 41), the European Hyperbaric Medicine Society (EHMS) recommended HBOT combined with medical therapy in patients with ISSNHL diagnosed within 2 weeks from the onset, or as the potential adjunct to steroids within 4 weeks from the onset, mainly in patients with severe and profound hearing loss. Several reports have shown improved hearing levels after HBOT in ISSNHL patients with initial therapy failure (5, 12, 17, 42–45). However, because of the small number of patients in the trials and methodological shortcomings, the AAO-HNS considered that the real benefit of HBOT for ISSNHL remains uncertain, so its use is not recommended in the guidelines, but it is reserved as an option (5). The small number of trials may be due to the limited availability of HBOT in many countries because of the cost ($600 to $700 per session in academic facilities in the United States) and the poor insurance coverage (5, 12).

A recent meta-analysis (12), including three observational studies and one randomized controlled trial, demonstrated that there were no significant differences in mean hearing gain between salvage ITS and salvage HBOT after failed primary systemic steroid treatment. In our experience, HBOT associated with systemic steroids as salvage therapy provided better, but not statistically significant, hearing recovery than the use of ITS associated with systemic steroids as salvage therapy. Indeed, the ratio of patients responding to therapy was 18.75% in group B vs. 16.7% in group C (*p* > 0.9), and one of the patients in group B experienced a complete hearing recovery. Moreover, patients in group B experienced a slightly better mean PTA gain than patients in group C (18.6 ± 21.1 dB vs. 15.7 ± 14.2 dB, *p* = 0.9). The hearing improvement after HBOT was maximal at 4000 Hz, and it was statistically significantly better than the gain of group C (*p* < 0.05). According to our findings, Cvorovic et al. (31) reported a better hearing improvement at high frequencies (2000 Hz) with the use of HBOT than with the use of ITS.

It is interesting to observe that in our study, the best audiological outcomes have been observed in patients of group A, who protracted only systemic steroids therapy because of refusal of salvage treatments or inability to receive them due to medical contraindications. In total, 10 of the 34 patients (29.4%) of group A showed a response to therapy, with a complete recovery in three cases. According to our findings, a recent retrospective study (33) reported a limited efficacy of salvage therapy with ITS or HBOT in hearing improvement. It must be considered that the high rate of spontaneous recovery (32%−65%) (5, 12, 46) could bias the treatment outcomes, so the results should be evaluated with caution.

Several factors have been suggested as a predictor of poor recovery, such as the presence of vertigo, descending audiometric configuration, and cardiovascular risk factors (diabetes and hyperlipidemia) (6, 33). We did not find any statistically significant differences between patients who responded to therapy and those who showed slight or no response regarding diabetes, hyperlipidemia, cardiovascular diseases, and audiogram shape. Conversely, vertigo was associated with worse outcomes in all three groups, and although the association was statistically significant only in group A (group A: *p* < 0.05, group B: *p* = 0.25, group C: *p* = 0.15), none of the patients with vertigo of the whole series recovered the hearing loss. The association of vertigo with worse outcomes suggests that vestibular symptoms may represent a poor prognostic factor in ISSNHL, regardless of the treatment. The higher incidence of vertigo in patients of group C (58%) and their worse pre-treatment PTA could be a reason for the lower hearing recovery observed in our cases treated with ITS.

Given the favorable natural history and inconclusive or modest benefit of the multiple treatment options, the AAO-HNS guidelines suggest involving patients in the decision for what, if any, treatment to undertake (5).

Our study has several limitations. This is a retrospective study based on medical charts. The distribution of the patients in the three groups, although based on their decision after adequate counseling, was not randomized, which can cause a selection bias. The different severities of hearing loss in the three groups could have influenced the audiological outcomes. Moreover, the small size of our series could have affected the statistical significance of the results, requiring future randomized controlled trials to definitively determine the impact of salvage treatments on hearing outcomes in patients with ISSNHL.

In conclusion, in our experience, ITS or HBOT associated with systemic steroids as salvage treatment did not show significant improvement in hearing outcomes. The evolution of ISSNHL, regardless of the treatment, remains unpredictable. The decision to perform salvage therapies should be based on the amount of persistent hearing loss following initial therapy, patient preference, as well as the risks of the treatment itself. Vertigo associated with hearing loss is a poor prognostic factor reflecting a severe injury of the labyrinths.

## Data availability statement

The raw data supporting the conclusions of this article will be made available by the authors, without undue reservation.

## Ethics statement

The studies involving humans were approved by AOU Cagliari (Ethics Committee protocol number 2022/5138). The studies were conducted in accordance with the local legislation and institutional requirements. Written informed consent for participation in this study was provided by the participants' legal guardians/next of kin.

## Author contributions

CM, FC, and RP designed the study, analyzed the data, and wrote and edited the manuscript. GC, SL, VM, SC, and MT collected and analyzed the data. All authors have read and agreed to the published version of the manuscript.
